# A Novel High-Throughput Assay for Islet Respiration Reveals Uncoupling of Rodent and Human Islets

**DOI:** 10.1371/journal.pone.0033023

**Published:** 2012-05-14

**Authors:** Jakob D. Wikstrom, Samuel B. Sereda, Linsey Stiles, Alvaro Elorza, Emma M. Allister, Andy Neilson, David A. Ferrick, Michael B. Wheeler, Orian S. Shirihai

**Affiliations:** 1 Department of Medicine, Boston University School of Medicine, Boston, Massachusetts, United States of America; 2 The Wenner-Gren Institute, Stockholm University, Stockholm, Sweden; 3 Departamento de Biologia, Facultad de Ciencias de la Salud, Universidad Andres Bello, Santiago, Chile; 4 Departments of Physiology and Medicine, University of Toronto, Toronto, Canada; 5 Seahorse Bioscience, North Billerica, Massachusetts, United States of America; 6 Tufts University School of Medicine, Boston, Massachusetts, United States of America; University of Bremen, Germany

## Abstract

**Background:**

The pancreatic beta cell is unique in its response to nutrient by increased fuel oxidation. Recent studies have demonstrated that oxygen consumption rate (OCR) may be a valuable predictor of islet quality and long term nutrient responsiveness. To date, high-throughput and user-friendly assays for islet respiration are lacking. The aim of this study was to develop such an assay and to examine bioenergetic efficiency of rodent and human islets.

**Methodology/Principal Findings:**

The XF24 respirometer platform was adapted to islets by the development of a 24-well plate specifically designed to confine islets. The islet plate generated data with low inter-well variability and enabled stable measurement of oxygen consumption for hours. The F1F0 ATP synthase blocker oligomycin was used to assess uncoupling while rotenone together with myxothiazol/antimycin was used to measure the level of non-mitochondrial respiration. The use of oligomycin in islets was validated by reversing its effect in the presence of the uncoupler FCCP. Respiratory leak averaged to 59% and 49% of basal OCR in islets from C57Bl6/J and FVB/N mice, respectively. In comparison, respiratory leak of INS-1 cells and C2C12 myotubes was measured to 38% and 23% respectively. Islets from a cohort of human donors showed a respiratory leak of 38%, significantly lower than mouse islets.

**Conclusions/Significance:**

The assay for islet respiration presented here provides a novel tool that can be used to study islet mitochondrial function in a relatively high-throughput manner. The data obtained in this study shows that rodent islets are less bioenergetically efficient than human islets as well as INS1 cells.

## Introduction

In type 2 diabetes, overt hyperglycemia occurs when insufficient amounts of insulin are secreted [1]. A growing body of evidence implies mitochondrial dysfunction in the pathophysiology of β-cell secretory failure [2]. β-cell mitochondria serve as the fuel integrator of the β-cell and generate signals for insulin secretion, the foremost being ATP. The respiratory chain complexes pump protons from the mitochondrial matrix to the intermembrane space, thus creating the proton gradient that upon its matrix reentry fuels oxidative phosphorylation at complex V (F1F0 ATP synthase) [3]. Alternatively, protons may return to the matrix through routes other than complex V, which results in oxygen consumption that is not coupled to ATP production; termed proton leak or uncoupling [4]. The level of uncoupling is an important biological phenomenon as it reflects bioenergetic efficiency. It was recently shown that INS-1 cells (rat insulinoma cell line) exhibit considerably high levels of uncoupling [5]. Seventy five percent of the respiration measured from INS-1 cells was reported to be uncoupled in comparison to 20% in myoblasts [5]. Considering the importance of mitochondrial ATP-production for insulin secretion this finding is certainly intriguing. To date, however, the uncoupled portion of respiration was not examined in the primary tissue: pancreatic islets.

While it is less commonly measured, oxygen consumption is arguably the most informative bioenergetic parameter [6]. At complex IV oxygen is consumed in a manner proportional to the proton extrusion. Thus, oxygen consumption (or respiration) directly reflects the flow of the electron transport chain. Recently it was shown that the respiratory profile of human islets prior to transplantation into animal models may predict the transplantation outcome [7], [8], [9]; i.e. islets with high levels of oxygen consumption were more likely to reverse diabetes. However, it is not yet clear what respiration parameter best predicts transplantation outcome; coupled or uncoupled Due to the scarcity of pancreatic islets (1–2% of the pancreas) performing experiments on isolated mitochondria from islets is not feasible. Several assays have been used over the years to measure oxygen consumption of intact isolated islets, first developed by Hellerstrom [10]. Despite providing high quality data when appropriately used, these assays are cumbersome to use and low throughput. Therefore, there is a need for the development of high throughput islet respirometry assays. Such an assay may be of great use both in basic research and in a clinical setting to evaluate islets prior to transplantation.

In this study we present the development and characterization of a novel high-throughput method for measuring coupling efficacy in islets. With this assay, we characterize the bioenergetic efficiency of rodent as well as human islets and make a comparison with INS-1 cells and C2C12 myotubes.

### Note

Throughout the paper oxygen consumption rate (OCR) and respiration are used as synonyms. Uncoupling (or proton leak) is defined as remaining respiration under a saturating concentration of the F1F0 ATP synthase inhibitor oligomycin.

## Results

### Increasing the Throughput of Islet Respirometry

**Figure 1:**
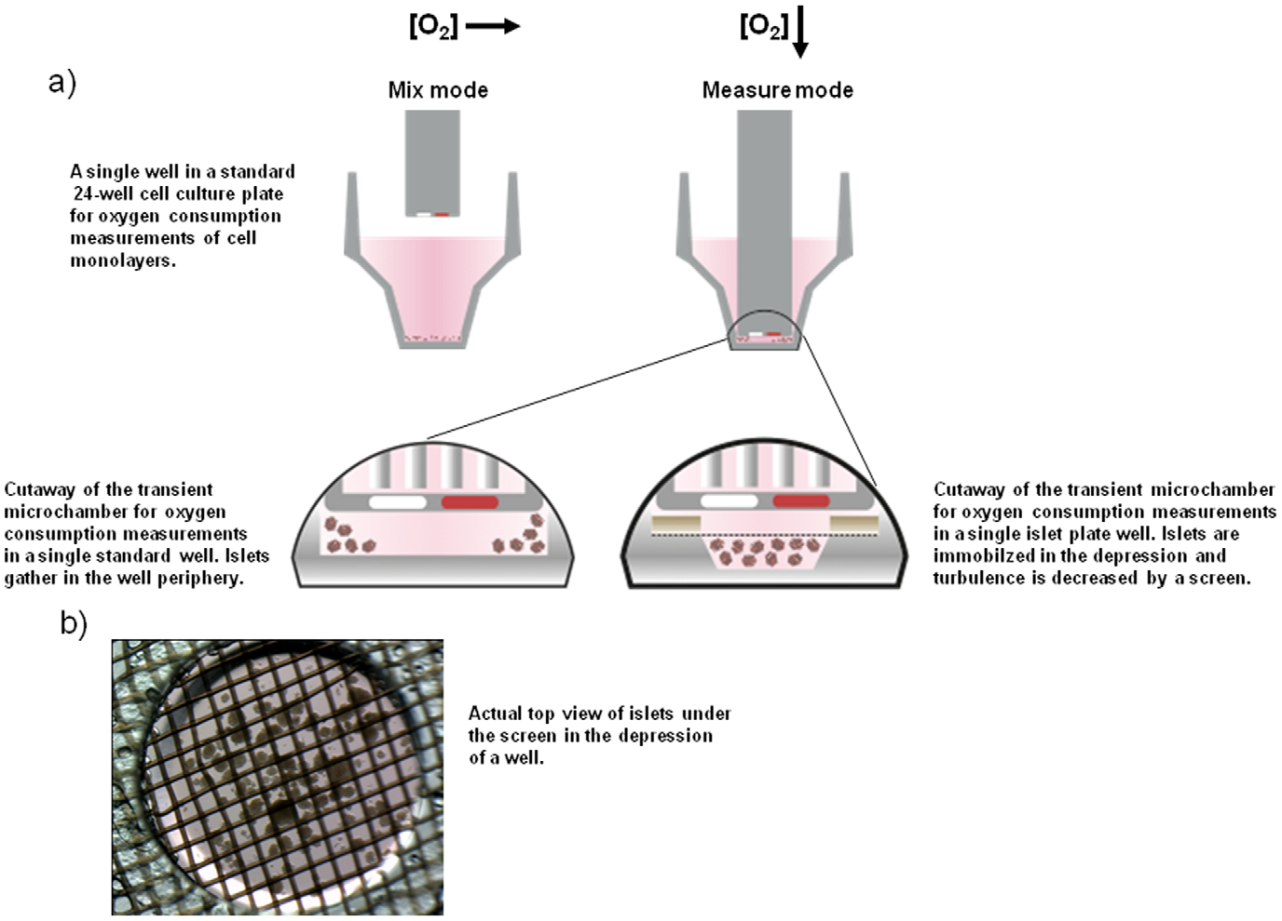
Measuring islet oxygen consumption in a high throughput format. A) Islet plate development. The XF24 measures oxygen consumption from monolayers of adherent cells in a 24 well cell culture plate format. Oxygen consumption is measured with probe heads coated with oxygen sensitive fluorophores that are optically read (marked white and red). During the “measure” mode a volume of only a few micro-liters is formed. In this minute volume oxygen tension rapidly drops which enables OCR calculations. In “mix” mode, the probe head moves up and down, reoxygenating the cells and exposing them to injected compounds. The wells with flat bottoms, originally designed for cell monolayers, proved inadequate for islets as they gathered in the well periphery, too far from the probe head (left panel). To address this problem, an islet plate with a central depression covered by a screen was developed. This trapped the islets while still maintaining sufficient media access (right panel). The screen consists of a polycarbonate ring attached to a nylon net with 50 µm pore size. B) Image of islet plate well. After experiments each well of the 24-well plate was imaged and the images were used for calculating OCR per islet and to measure islet diameter. The islet images were then used to obtain an exact islet count per well and to measure islet diameter. C) Flat bottom (V7) plates are not suitable for islets. 70 islets were seeded per well. Traces recorded from separate wells in a plate are shown. Note the large data variation and that subsequent to 20 mM glucose injection the OCR only increase in one well out of four. D) Islet plate reduces data variability. As islet seeding could vary and thus the absolute OCR, data from each well was often normalized to its initial steady state values before any compound was injected. Traces from islets in 3 mM and 20 mM glucose are shown with absolute (left) and normalized rates (right); note the stability over 2.5 hours. Subsequent to 20 mM glucose injection (after 1^st^ data point) the OCR increased in all the wells. Islets that stay in 3 mM glucose do not display any major change in OCR. E) Initial measurements are unstable. OCR in the islet plate showed an initial drift during the first 1–2 measurements (dashed square), therefore these data points where always omitted. F) Experimental set up – bioenergetic principles. Respiration recorded may be deciphered by using drugs acting on the mitochondrial inner membrane/complexes. Oligomycin blocks complex V and remaining respiration represents the proton leak. Rotenone/Myxothiazol blocks complexes I/III and remaining respiration is non-mitochondrial. FCCP stimulation shows maximal respiratory capacity. Nutrient stimulation (glucose) may be used in addition to these drugs to study the effect of nutrient metabolism.

To develop a higher throughput islet respirometry assay we built upon the existing respirometry platform originally designed for adherent cells, XF24 (Seahorse Bioscience, Billerica, MA). The XF24 measures oxygen consumption from monolayers of adherent cells in a 24-well cell culture plate format [11]. The plates used for cell monolayers proved inadequate for non-adherent islets as they gathered in the periphery of the well due to turbulence from pipetting and probe head movement (Fig. 1A). This produced data with high variability and unreliable response to stimuli, such as glucose (Fig. 1B). To solve this, we designed a plate that enclosed the islets during an experiment (Fig. 1A). The islets are kept in a depression in the middle of the well that keeps them in close proximity to the probe head and a screen is used to protect them from turbulence. The screen consists of a polycarbonate ring attached to a nylon net with a 50 µm pore size. After an experiment, which could last up to 5 h, the islet plate was imaged and the islets were harvested for protein or other downstream assays. The islet images were then used to obtain an exact islet count per well and to measure islet diameter for normalization.

### Measuring Islet Oxygen Consumption

On the day of experiment islets were transferred by pipetting to 50 ml conical tubes with warm assay media containing 3 mM glucose, 0.8 mM Mg^2+^, 1.8 mM Ca^2+^, 143 mM NaCl, 5.4 mM KCl, 0.91 mM NaH2PO4, Phenol red 15 mg/ml (Seahorse Bioscience); and maintained in this media throughout the experiment. As the experiment is performed in an environment of atmospheric CO_2_, we avoided using a bicarbonate based buffer. In order to prevent islets from becoming sticky, the media was supplemented with 1% FBS. After one wash islets were resuspended in 2 ml of media. 50 to 80 islets were plated into the islet plate by pipetting 2×50 µl of stirred islet mix into each of the 20 wells, loaded previously with 400 µl of media. Four wells were kept empty as controls in every experiment. Screens were carefully put on top of the depression of all wells with tweezers. To avoid bubble formation in the net, screens were pre-wetted with media. The islet plate was then incubated for 60 minutes at 37°C before it was loaded into the XF24 respirometry machine (Seahorse Bioscience). During this time a cartridge with the injection compounds (50 µl/port) used during the experiment was prepared and the machine programmed.

Oxygen consumption rates (OCR) were measured at basal glucose levels as well as with drugs acting on the respiratory chain: oligomycin (F1FOATPsynthase inhibitor; 5 µM), FCCP (uncoupler: 1 µM), rotenone (complex I inhibitor; 5 µM) and antimycin A (complex III inhibitor; 5 µM); all from Sigma; and myxothiazol (complex III inhibitor; 5 µM; Calbiochem, La Jolla, CA). In total, the preparation time before starting the measurements was 2–3 h.

### Islet Size Measurements and Absolute Oxygen Consumption Rates Per islet

To measure islet size, bright field images were taken of each islet well immediately after the experiment with an Olympus SZX16 stereo microscope. Islet diameter was then measured with Metamorph image analysis software (Molecular Devices, Sunnyvale, CA). Absolute oxygen consumption rates (OCR) per islet could then be derived by counting the number of islets per well.

### Data Normalization

The islet plate provided absolute OCR (pmoles O2/min) which could vary between wells as the number of islets seeded in different wells could vary (Fig. 1C). To adjust for the variation in islet number OCR of each well was normalized to the initial rates under basal conditions (Fig. 1C). This produced data with less variability that was suitable for comparing different experimental conditions. An initial drift in OCR was typically observed in the first 1–2 measurements until steady state was reached (Fig. 1D). Therefore, these initial data points were always disregarded.

### Measuring Coupling Efficacy

**Figure 2:**
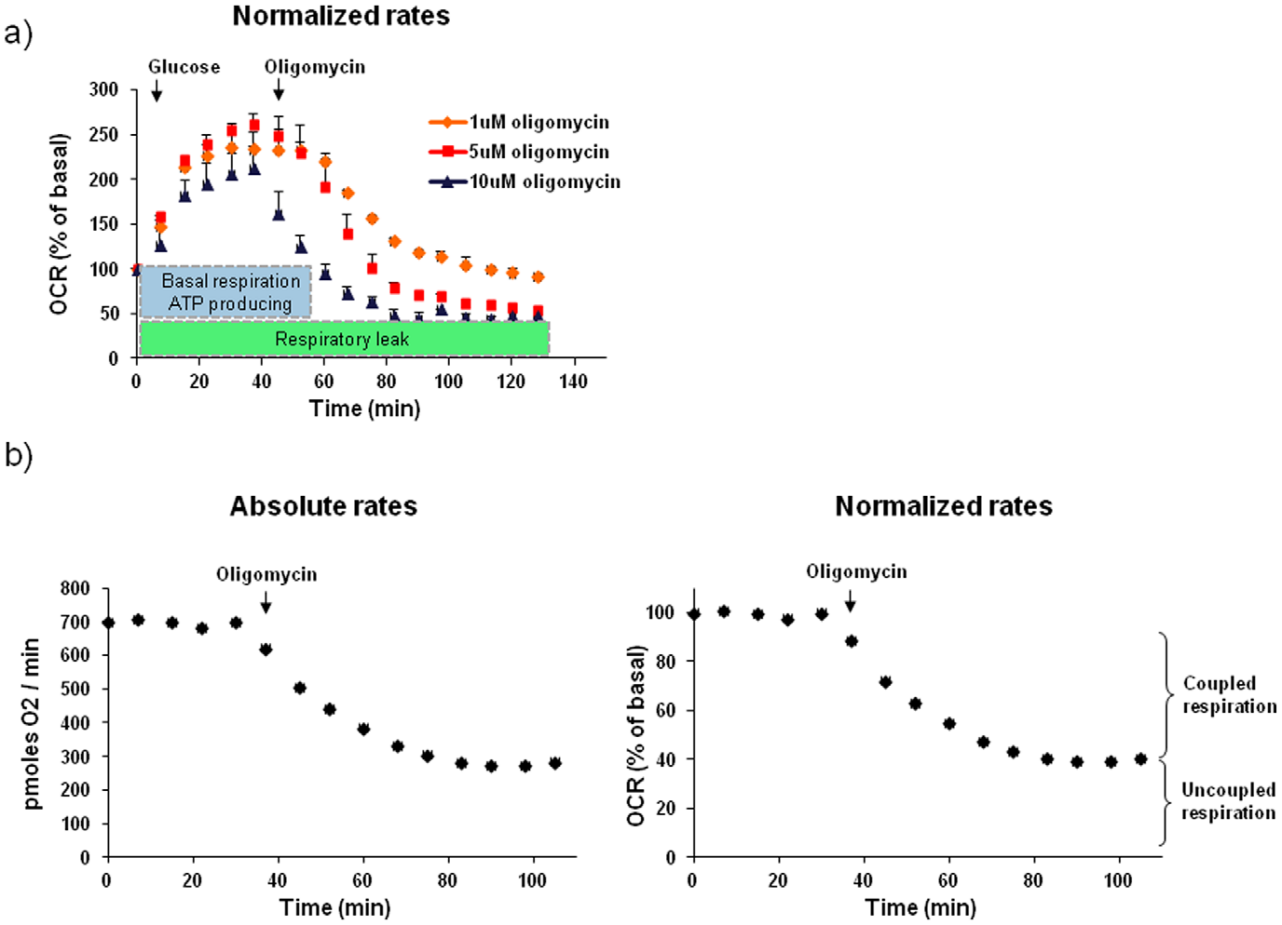
Measuring coupling efficacy. A) Oligomycin titration. The F1F0 ATP synthase inhibitor oligomycin was added after 20 mM glucose as indicated. Note that 5 µM oligomycin has a similar effect as 10 µM. B) Experimental setup to measure uncoupling. A few data points are recorded under basal conditions (3 mM glucose) followed by injection of oligomycin. The remaining OCR under oligomycin represents respiration that is not coupled to ATP synthesis (uncoupling). Data is shown both as absolute rates (left) and normalized to initial values (right). C) FCCP increase OCR under oligomycin. To verify the capacity of oligomycin to reveal uncoupling, islets were treated with FCCP (1 µM) prior to oligomycin. Note the increase in OCR under oligomycin representing maximal uncoupled respiration. D) Norepinephrine activated brown adipocytes show higher respiration under oligomycin. Basal measurement was followed by injection of control media or media containing 1 µM norepinephrine which activates uncoupling. Note the increase in OCR under oligomycin indicative of uncoupling. E) Oligomycin does not alter non-mitochondrial respiration. OCR was first measured with no drugs present followed by injection of only oligomycin (OM) or both oligomycin and rotenone/myxothiazol (Rot) (5 µM of both). Later, islets that were only injected with oligomycin at first also received rotenone/myxothiazol. Note that there is no difference in non mitochondrial respiration (under rotenone/myxothiazol) between the different treatments indicating that oligomycin does not alter non mitochondrial respiration.

Using isolated mitochondria to examine uncoupling is common; however it is not feasible with islets. E.g. a standard mitochondrial isolation from 1×10^6^ cells would require more than 6000 islets isolated from 30–40 mice (assuming each islet has 1500 cells). To address this issue we developed an approach to assess uncoupling of intact islets based on oligomycin, a specific inhibitor of the F1F0ATPsynthase [12]. The basic experimental algorithm was as follows (Fig. 2B). First, basal respiration levels were measured repeatedly until stable. Then, in order to determine the level of uncoupling, oligomycin was added at a saturating concentration (Fig. 2A). Oligomycin caused a decrease in respiration that reached a steady-state within 30–45 min (Fig. 2A). As absolute rates of oxygen consumption varied depending on islet seeding number, each well was normalized to its initial basal rates when only relative measurements were needed (Fig. 2B). To determine what fraction of total respiration is uncoupled, only relative values are needed.

To verify that measurements under oligomycin indeed reflect uncoupling the uncoupler FCCP was added prior to oligomycin (Fig. 2C). This resulted in higher rates of islet respiration under oligomycin, representing maximal uncoupled respiration. In order to confirm that we could measure physiological endogenous uncoupling we examined brown adipocytes which uncouple via uncoupling protein 1 (UCP1) in response to adrenergic stimulation [13]. It was evident that norepinephrine stimulated respiration was higher under oligomycin, again verifying the assays ability to measure uncoupling (Fig. 2D). Further, in order to measure non-mitochondrial respiration, rotenone (complex I inhibitor) and myxothiazol or antimycin A (complex III inhibitor) were added to the islets in the end of each experiment. Data from multiple experiments showed an average of 8.1% of the basal respiration remaining under rotenone and myxothiazol/antimycin A (n = 25). To examine if oligomycin affected the non-mitochondrial respiration, an experiment was performed where these compounds were added before oligomycin (Fig. 2E). There was no further decrease in OCR when oligomycin was added on top of rotenone and myxothiazol/antimycin A. Thus, only a minor part of the uncoupling measured under oligomycin is non-mitochondrial and is not influenced by the oligomycin treatment itself.

### Islets have High Levels of Uncoupling

**Figure 3:**
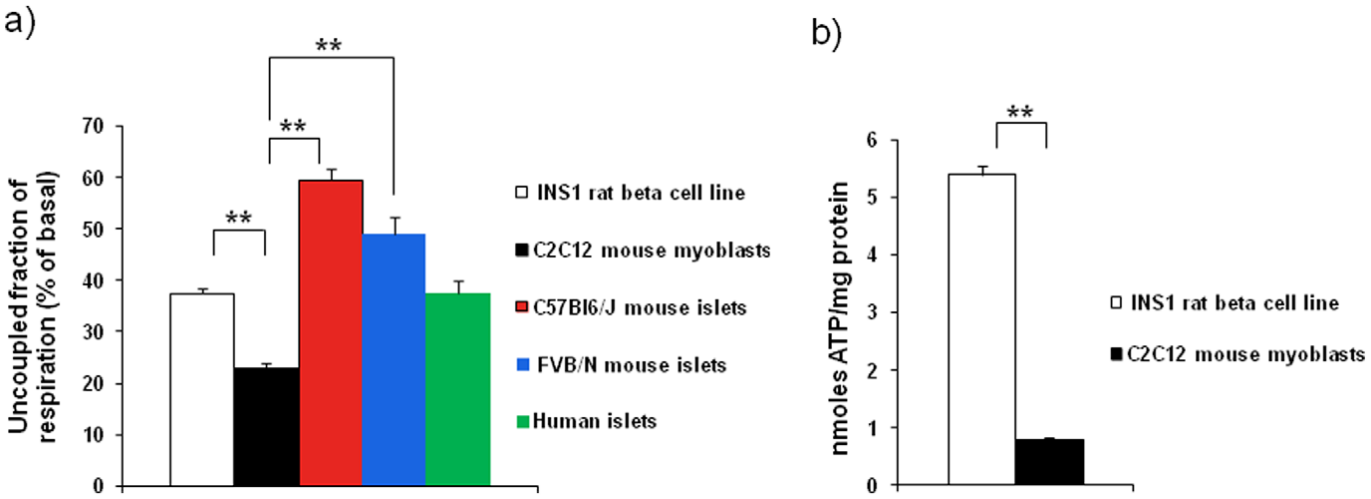
Islets are highly uncoupled; mice islets more than human. A) Uncoupling of wild type mouse islets, INS-1 cells, C2C12 myotubes and human islets. The graph presents a summary of multiple experiments where OCR under oligomycin (defined as uncoupling) was measured until stable. Note that uncoupled OCR is significantly higher in the mouse islets and that the C2C12 myotubes show the lowest uncoupled OCR. N = 29 (INS-1), 20 (C2C12), 35 (C57Bl6/J islets), 14 (FVB/N islets), 8 (Human islets). **indicates p<0.01. B) Comparison of total ATP levels in INS-1 cells vs. C2C12 myotubes.

To date there are no reports on the level of uncoupling in intact islets. In this study we examined islets from mice of two different genetic backgrounds; C57Bl6/J and FVB/N. Islets from C57Bl6/J mice exhibited a basal level of uncoupling (under oligomycin) of 59% as compared to basal respiration (100%, no oligomycin) (Fig. 3A). In contrast, FVB/N islets showed slightly lower levels of uncoupling: 49%. In clonal INS-1 cells and C2C12 myotubes uncoupling was measured to 38% and 23% respectively. Moreover, islets from a cohort of non diabetic donors were also examined. Interestingly, the level of uncoupling was significantly lower in the human islets as compared to the rodent islets.

As higher levels of uncoupling may lead to less mitochondrial ATP production, we compared total ATP levels of INS-1 cells and C2C12 myotubes (Fig. 3B). Total ATP levels were considerably higher in INS-1 cells.

**Figure 4:**
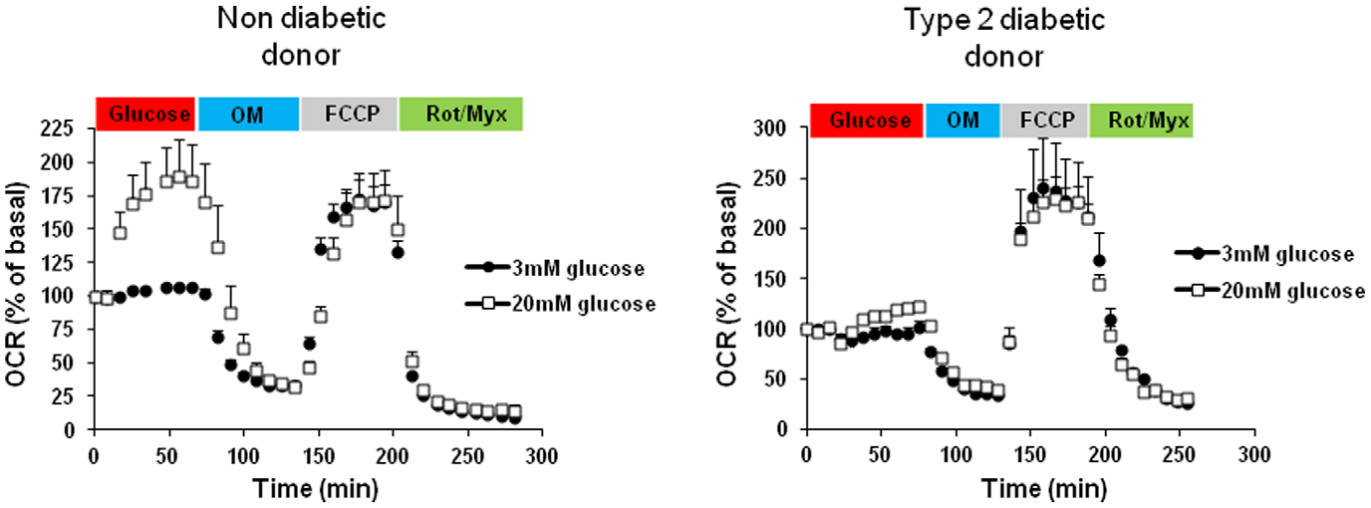
Respiration of a small cohort of human Type 2 diabetic islets. Each graph represents islets from one patient (n = 4−5). Drugs added during the experiment are indicated. Type 2 diabetic donor: Male age 52, weight 100 kg, BMI 31.9, Caucasian. Non diabetic donor: Male age 32, weight 69 kg, BMI 24.7, Caucasian.

Moreover, we examined the level of uncoupling of islets from three Type 2 diabetic patients (Fig. 4). These islets presented with similar levels of uncoupling as the healthy control islets (Fig. 3A) although the n was not high enough for a meaningful statistical comparison.

## Discussion

In this study we present the development of a novel islet respirometry assay and use it to examine the bioenergetic efficiency of islets. We first show that the assay can measure islet oxygen consumption in a reliable and high throughput manner. By using the F1F0 ATP synthase blocker oligomycin we could distinguish between coupled and uncoupled respiration in intact cells and islets. Wild type rodent islets from mice with two different genetic backgrounds exhibited high levels of uncoupling as compared to clonal INS-1 cells and C2C12 myotubes. In comparison with rodent islets, human islets showed lower levels of uncoupling, indicating higher bioenergetic efficiency.

### High Throughput Islet Respirometry

In this study we developed a novel high throughput method for assessing islet respiration (Fig. 1). The vast majority of cellular oxygen consumption is mitochondrial and occurs at complex IV in the respiratory chain as protons are pumped out to the intermembrane space [3]. Oxygen consumption is possibly the most informative mitochondrial parameter [6] as it directly reflects the flow of the respiratory chain. Several assays for measuring islet oxygen consumption have been used over the years and were recently reviewed in detail [9]. Perhaps the most intriguing data is coming from the pioneering studies of Sweet *et al* and Papas *et al* who reported that human islets with high oxygen consumption rates are more suitable for transplantation [7], [8], [9], [14]. Strikingly, a model incorporating islet oxygen consumption per DNA content was able to predict transplantation outcome (diabetes reversal) with sensitivity of 93% and specificity of 94% [14]. It was suggested that oxygen consumption may be useful to predict marginal mass for diabetes reversal, an important matter because of the scarcity of islet donors.

In principal, respiration assays have utilized either electrodes or fluorescent probes to report on oxygen tension in the media. Current assays that measures islet oxygen consumption provide quality data [9], [15], [16], [17], [18]. Some have the advantage of using media flow-through and can sample in and outflow, thus, for example, enabling insulin secretion measurements. This is a feature that our islet respirometry assay lacks. However, the flow-through methods can only run one sample per experiment and requires a high level of training in this technique. Alternatively, multiwell plates coated with oxygen sensitive fluorescent probes have been used for higher throughput analysis of oxygen consumption [19], [20]. Although these assays are higher throughput, the coated plates are designed for cell monolayers and only provide relative data. Therefore, up to now there has been a lack of high throughput and user friendly assays that allow for the study of non-adherent cells that are not arranged in a monolayer. The new respirometry assay presented here may not only be of value to basic islet research, but may also prove important clinically to evaluate islet quality prior to transplantation as it may readily be used in different islet transplantation centers.

### Oligomycin Unmasks Uncoupling

In principle, respiration represents both protons used for ATP production as well as proton leak [21], [22]. Thus, a change in oxygen consumption between samples has a dual interpretation; it can represent coupled or uncoupled respiration, the latter of which may be interpreted as increased bioenergetic inefficiency. To reveal the level of uncoupling the F1F0 ATP synthase inhibitor oligomycin was used (Fig. 2). As oligomycin is a mitochondrial toxin, caution must be practiced when interpreting its effects. To start with, it has been shown that proton leak rates in isolated mitochondria increase with Δψ_m_ [4]. A possible caveat of using oligomycin may therefore be its Δψ_m_ hyperpolarizing effect. In mouse β-cells, the relative change in Δψ_m_ with oligomycin was measured to ∼6 mV [23]. To what degree Δψ_m_ hyperpolarization influences our measurements of uncoupling is not clear as the absolute Δψ_m_ of intact cells is not possible to measure. However even if the Δψ_m_ changes would be in the upper part of scale, the changes in proton leak rate upon fuel stimulation would only be in the order of 10–15% [4].

### Islets are Highly Uncoupled

Proton leak is a major contributor to energy homeostasis [24]. In principle, the level of uncoupling is of interest as it reflects the bioenergetic efficiency of the cell. In this study we show that mouse islets are highly uncoupled, ∼50–60% of the basal respiration remains under oligomycin (Fig. 3). Of this ∼8% may represent non-mitochondrial respiration. To the best of our knowledge, this is the first characterization of islet uncoupling. In comparison, a recent INS-1 cell study measured uncoupling to 75% [5]. This is in contrast with our measurements of uncoupling in INS-1 cells where the level of uncoupling was 38% (Fig. 3). This difference is most likely not due to methodology differences as our measurements of uncoupling in C2C12 myotubes (∼22%) were similar to Affourtit and colleagues results [5]. We suspect that this is more likely due to the known diversity in the behavior of different clones of INS-1 cells.

The cohort of human islets showed significantly lower levels of uncoupling as compared to both types of mouse islets (Fig. 3). This also appeared to be the case for human diabetic islets (Fig. 4). That is, human islets appear more bioenergetically efficient. These data add to previous reports on differences between rodent and human islets, e.g. human islets exhibit a different distribution of alpha cells [25]. The reason for divergence in uncoupling is not clear but may be of importance for insulin secretion signaling. At a certain fuel level (e.g. glucose), the mitochondrial ATP output from human β-cell mitochondria may be higher in human islets. It would certainly be of interest to expand the cohort of human diabetic islets.

Somewhat counterintuitive, ATP levels were significantly higher in INS-1 cells as compared to C2C12 myotubes (Fig. 3B). Thus, although the respiratory leak was higher, the energetic status of the INS-1cell was not compromised. This may reflect the different cellular roles of beta cells and myocytes; while the former serves as a “fuel sensor” the latter serves as a “fuel utilizer”. Recently, it was shown that uncoupling protein 2 (UCP2) does not contribute to mitochondrial uncoupling in beta cells in intact islets [26]. Thus, the question on what regulates mitochondrial uncoupling in islets is still open.

## Materials and Methods

### Experimental Animals

10 to 14-week-old male wild type C57BL6/J (Jackson lab., Bar Harbor, ME) and FVB/N mice (Charles River Canada, Montreal, Canada) were used. Animals were fed normal chow and maintained under controlled conditions (19–22°C and a 14∶10-h light-dark cycle) until death by CO_2_ asphyxiation. All procedures were performed in accordance with the Boston University Institutional Guidelines for Animal Care (IACUC no. 1104) in compliance with U.S. Public Health Service Regulation.

### Islet Isolation and Culture

Islets of Langerhans were isolated as previously described by collagenase injection into the bile duct [27]. After isolation, intact islets were cultured overnight in RPMI-1640 culture media supplemented with 10 mM glucose, 10% FBS, 100I U/ml penicillin, and 100 µg/ml streptomycin; all from Invitrogen (Carlsbad, CA).

Human islets were isolated from 10 cadaveric donors at facilities at Massachusetts General Hospital (Boston, MA) and at facilities affiliated with NDRI (National Disease Research interchange). Islets were isolated with standard methods and shipped overnight on ice. Of 10 donors, 9 were male; their ages ranged from 20 to 62 years (average 45 years). The cold ischemia time ranged from 2 to 27 h (average 11 h). Purity of the islet preparations ranged from 70 to 93% (average 83%), however upon arrival islets were further purified by handpicking. Islets were placed in the same medium described above and incubated overnight at 37°C and 5% CO_2_. Informed written consent was obtained from the relatives of the donors. IRB approval details:

Name of committee: BUMC IRB

IRB Coordinator: Rosana B. Schomer

IRB Institution: Boston University

560 Harrison Avenue, Suite 300

Boston, MA 02118

Phone: 617-414-1320 Fax: 617-638-7234

E-mail: roz@bu.edu

Web: www.bumc.bu.edu/irb

IRB APPROVAL details:

Type: Exempt

Approval number: H-27516

Approval date Dec 4th 2008

### Cell Culture and Oxygen Consumption Measurements

Clonal INS-1 cells were cultured in RPMI-1640 media supplemented with 10% FBS, 10 mM HEPES buffer, 1 mM pyruvate, 50 µM 2-ß-mercaptoethanol, 50 U/ml penicillin and 50 µg/ml streptomycin. 10^5^ cells were seeded per V7 cell culture plate (Seahorse Bioscience) well the day prior to experiment.

The murine cell line C2C12 myoblasts (American Type Culture Collection) were maintained in DMEM media with 4.5 g/liter glucose, 10% FBS, 100 U/ml penicillin and 100 µg/ml streptomycin. To induce differentiation, media was replaced with DMEM containing 2% horse serum when cells reached 80–85% confluency. Experiments were performed on differentiated C2C12 myotubes after 4 days.

Mouse brown adipocytes were isolated and cultured as previously described in detail [28]. In brief, brown adipose tissue from three 3 week old C57BL6/J male mice was digested by collagenase, washed and resuspended in 6 ml of culture medium. A volume of 100 µl was seeded into each well of a V7 cell culture plate followed by cell attachment for 1 h and thereafter addition of 150 µl media. Media was changed the day after seeding and after that every second day until preadipocytes had differentiated into brown adipocytes after 7days of culture.

For oxygen consumption measurements, INS-1, C2C12 and brown adipocytes were switched to assay media (3 mM glucose but no FBS), and further processed as the islet plate (see Results).

### ATP Measurements

ATP measurements were performed using the ATP Bioluminescence Assay Kit CLS II (Roche Diagnostics, Mannheim, Germany). Both INS-1 and differentiated C2C12 cells were diluted to 10^6^ cells/ml in PBS. The standard protocol from Roche was followed and luminescence readings were taken on the Infinite® M1000 microplate reader (Tecan, Männedorf, Switzerland). Calculated total nmoles of ATP was normalized to total protein levels obtained using a BCA Protein Assay Kit (Pierce, Rockford, IL).

### Statistical Analysis

Data are given as means ± SEM. Two-tailed, unpaired, or paired Student’s *t* tests were used to compare data sets. P<0.05 was considered significant.

## References

[1] Leahy JL (2008). Mary, Mary, quite contrary, how do your beta-cells fail?. Diabetes.

[2] Mulder H, Ling C (2009). Mitochondrial dysfunction in pancreatic beta-cells in Type 2 diabetes.. Mol Cell Endocrinol.

[3] Mitchell P (1976). Vectorial chemistry and the molecular mechanics of chemiosmotic coupling: power transmission by proticity.. Biochem Soc Trans.

[4] Brand MD, Brindle KM, Buckingham JA, Harper JA, Rolfe DF (1999). The significance and mechanism of mitochondrial proton conductance.. Int J Obes Relat Metab Disord.

[5] Affourtit C, Brand MD (2008). Uncoupling protein-2 contributes significantly to high mitochondrial proton leak in INS-1E insulinoma cells and attenuates glucose-stimulated insulin secretion.. Biochem J.

[6] Will Y, Hynes J, Ogurtsov VI, Papkovsky DB (2006). Analysis of mitochondrial function using phosphorescent oxygen-sensitive probes.. Nat Protoc.

[7] Papas KK, Colton CK, Nelson RA, Rozak PR, Avgoustiniatos ES (2007). Human islet oxygen consumption rate and DNA measurements predict diabetes reversal in nude mice.. Am J Transplant.

[8] Sweet IR, Gilbert M, Jensen R, Sabek O, Fraga DW (2005). Glucose stimulation of cytochrome C reduction and oxygen consumption as assessment of human islet quality.. Transplantation.

[9] Sweet IR, Gilbert M, Scott S, Todorov I, Jensen R (2008). Glucose-stimulated increment in oxygen consumption rate as a standardized test of human islet quality.. Am J Transplant.

[10] Hellerstrom C (1966). Oxygen consumption of isolated pancreatic islets of mice studied with the cartesian-diver micro-gasometer.. Biochem J.

[11] Wu M, Neilson A, Swift AL, Moran R, Tamagnine J (2007). Multiparameter metabolic analysis reveals a close link between attenuated mitochondrial bioenergetic function and enhanced glycolysis dependency in human tumor cells.. Am J Physiol Cell Physiol.

[12] Grover GJ, Marone PA, Koetzner L, Seto-Young D (2008). Energetic signalling in the control of mitochondrial F1F0 ATP synthase activity in health and disease.. Int J Biochem Cell Biol.

[13] Cannon B, Nedergaard J (2004). Brown adipose tissue: function and physiological significance.. Physiol Rev.

[14] Papas KK, Colton CK, Qipo A, Wu H, Nelson RA (2010). Prediction of marginal mass required for successful islet transplantation.. J Invest Surg.

[15] Longo EA, Tornheim K, Deeney JT, Varnum BA, Tillotson D (1991). Oscillations in cytosolic free Ca2+, oxygen consumption, and insulin secretion in glucose-stimulated rat pancreatic islets.. J Biol Chem.

[16] Ortsater H, Liss P, Lund PE, Akerman KE, Bergsten P (2000). Oscillations in oxygen tension and insulin release of individual pancreatic ob/ob mouse islets.. Diabetologia.

[17] Jung SK, Gorski W, Aspinwall CA, Kauri LM, Kennedy RT (1999). Oxygen microsensor and its application to single cells and mouse pancreatic islets.. Anal Chem.

[18] Doliba NM, Qin W, Vatamaniuk MZ, Buettger CW, Collins HW (2006). Cholinergic regulation of fuel-induced hormone secretion and respiration of SUR1−/− mouse islets.. Am J Physiol Endocrinol Metab.

[19] Fraker C, Timmins MR, Guarino RD, Haaland PD, Ichii H (2006). The use of the BD oxygen biosensor system to assess isolated human islets of langerhans: oxygen consumption as a potential measure of islet potency.. Cell Transplant.

[20] MacGregor RR, Williams SJ, Tong PY, Kover K, Moore WV (2006). Small rat islets are superior to large islets in in vitro function and in transplantation outcomes.. Am J Physiol Endocrinol Metab.

[21] Boudina S, Sena S, Theobald H, Sheng X, Wright JJ (2007). Mitochondrial energetics in the heart in obesity-related diabetes: direct evidence for increased uncoupled respiration and activation of uncoupling proteins.. Diabetes.

[22] Brand MD, Pakay JL, Ocloo A, Kokoszka J, Wallace DC (2005). The basal proton conductance of mitochondria depends on adenine nucleotide translocase content.. Biochem J.

[23] Wikstrom JD, Katzman SM, Mohamed H, Twig G, Graf SA (2007). beta-Cell mitochondria exhibit membrane potential heterogeneity that can be altered by stimulatory or toxic fuel levels.. Diabetes.

[24] Rolfe DF, Brand MD (1996). Contribution of mitochondrial proton leak to skeletal muscle respiration and to standard metabolic rate.. Am J Physiol.

[25] Brissova M, Fowler MJ, Nicholson WE, Chu A, Hirshberg B (2005). Assessment of human pancreatic islet architecture and composition by laser scanning confocal microscopy.. J Histochem Cytochem.

[26] Robson-Doucette CA, Sultan S, Allister EM, Wikstrom JD, Koshkin V (2011). {beta}-Cell Uncoupling Protein 2 Regulates Reactive Oxygen Species Production, Which Influences Both Insulin and Glucagon Secretion.. Diabetes.

[27] Lacy PE, Kostianovsky M (1967). Method for the isolation of intact islets of Langerhans from the rat pancreas.. Diabetes.

[28] Cannon B, Nedergaard J (2001). Cultures of adipose precursor cells from brown adipose tissue and of clonal brown-adipocyte-like cell lines.. Methods Mol Biol.

