# Correction: A Novel High-Throughput Assay for Islet Respiration Reveals Uncoupling of Rodent and Human Islets

**DOI:** 10.1371/annotation/f21efac9-2906-4a2f-ad22-befcb7714dd0

**Published:** 2013-12-06

**Authors:** Jakob D. Wikstrom, Samuel B. Sereda, Linsey Stiles, Alvaro Elorza, Emma M. Allister, Andy Neilson, David A. Ferrick, Michael B. Wheeler, Orian S. Shirihai

Due to errors introduced during the production process, Figure 1 and Figure 2 are incomplete. The full, correct figures can be found here:

Figure 1: http://plosone.org/corrections/pone.0033023.g001.cn.tif

Figure 2: http://plosone.org/corrections/pone.0033023.g002.cn.tif

